# Regional, socioeconomic, and health determinants of physical fitness in school children: insights from a National Olympic Fitness Project

**DOI:** 10.1093/eurpub/ckag016

**Published:** 2026-02-26

**Authors:** Pavol Ďurček, Libuša Nechalová, Milan Špánik, Branislav Bleha, Viktor Bielik

**Affiliations:** Department of Economic and Social Geography, Demography and Territorial Development, Faculty of Natural Sciences, Comenius University in Bratislava, Bratislava, Slovakia; Department of Biological and Medical Science, Faculty of Physical Education and Sports, Comenius University in Bratislava, Bratislava, Slovakia; Department of Olympism, Slovak Olympic and Sports Committee, Bratislava, Slovakia; Department of Economic and Social Geography, Demography and Territorial Development, Faculty of Natural Sciences, Comenius University in Bratislava, Bratislava, Slovakia; Department of Biological and Medical Science, Faculty of Physical Education and Sports, Comenius University in Bratislava, Bratislava, Slovakia

## Abstract

This study explores regional differences in physical fitness among Slovak school children, focusing on predictors such as school infrastructure, socioeconomic factors, life expectancy, and body mass index (BMI). It aims to provide a comprehensive, gender-specific analysis of systemic and environmental determinants influencing physical fitness. Data from the Slovak Olympic and Sports Committee’s National Physical Fitness Project during the 2023–24 academic year included 42 741 students aged 12–16 from all eight regions of Slovakia. Physical fitness was assessed using the EUROFIT test battery. Predictors included regional socioeconomic indicators (e.g. unemployment, education), health determinants (e.g. BMI, life expectancy), and school infrastructure. Factor analysis reduced multicollinearity, and regression models were stratified by gender across Grades 6–7 and 8–9. BMI emerged as the strongest negative predictor of physical fitness (−0.64 for younger boys, −0.41 for older boys). Life expectancy positively influenced fitness (up to 0.50 for older boys). Higher knowledge capital was associated with lower fitness, especially in younger students (−0.26 for boys and −0.15 for girls). Social vulnerability and gender equality had a pronounced negative impact on girls (−0.68), while insufficient school infrastructure reduced fitness in older boys (−0.44). The regression models explained 58%–71% of variance in physical fitness (*R*^2^ = 0.58–0.71). This study highlights the multifactorial nature of physical fitness disparities among school children, emphasizing the roles of BMI, socioeconomic factors, and school infrastructure. The findings underscore the need for targeted interventions addressing systemic inequalities to improve physical fitness and health equity in school-aged populations.

## Introduction

The obesity pandemic among school children poses significant challenges to global health, with rising rates linked to sedentary lifestyles [1]. Schools play a key role in promoting physical activity through physical education (PE), sports, and active play, with research showing that the number of hours spent in these activities strongly affects children’s fitness and health [2]. An increased number of PE classes has been associated with better weight management, improved cardiovascular health, and enhanced physical and academic performance [3]. The evidence supports school-based interventions in preventing childhood obesity and improving physical fitness, thereby fostering healthier children [4]. However, more evidence on how the physical fitness of school children contributing to general health is associated with demographic factors and socioeconomic conditions is needed.

Although the importance of the school environment and the specific role of school infrastructure and school types in influencing physical activity among school children is well recognized, there is a noted research gap regarding the impact of sociodemographic variables on health risks [5]. Additionally, socioeconomic inequalities may play a crucial role, as regions with higher household income often have better resources and, presumably, more opportunities for physical activity and better physical fitness [6]. However, the results are controversial [7] while social and economic factors are in most of the studies combined [8]. Furthermore, the average life expectancy, as well as cause-specific and all-cause mortality in midlife, were associated with physical activity during childhood and adolescence [9]. Understanding how these factors interact is essential for addressing disparities in physical fitness and health outcomes among school children.

Therefore, this study aims to compare regional differences in physical fitness among school children in Slovakia by examining the influence of key predictors, including school infrastructure, socioeconomic factors, average life expectancy, and body mass index (BMI). It further explores gender-specific differences by constructing separate models for boys and girls across different school grades. Using unique data from the Slovak Olympic and Sports Committee’s (OLOV) national project, the study offers new insights into systemic factors influencing physical performance and health outcomes.

## Methods

### Study design and participants

This study analyzed data from a national physical fitness assessment conducted during the 2023–24 academic year across all eight regions of Slovakia. The research was part of the National Physical Fitness Project of the OLOV, which invited all primary schools to participate [10]. A total of 42 741 students from 465 public schools, representing ∼20% of the eligible population, were included in the study. The target group consisted of students in Grades 6–9 (ages 12–16 years). Inclusion criteria required complete data on physical fitness and BMI for participation in the analysis. The study was approved by the Ethics Committee of the Faculty of Physical Education and Sport, Comenius University (No. 06/2024), the Slovak Olympic and Sports Committee, and participating school administrators. The research adhered to the criteria and ethical standards established in the field of sport and exercise science [11]. All participants were apprised of the study’s aims, and informed written agreement was secured from their legal guardians.

### Physical fitness assessment

Physical fitness was assessed using the standardized EUROFIT test battery, a widely recognized tool applied across European countries [12] and a nationally approved standard within the school curriculum in Slovakia. The test battery comprised bent arm hang, standing broad jump, 10 × 5 m shuttle run, sit-ups, 20 m endurance shuttle run, and medicine-ball throw. The testing has been implemented annually since 2018 and is coordinated by the Slovak Olympic and Sports Committee. Data for this study were collected during the 2023/2024 academic year in regular PE classes by 588 university graduates in PE. Moreover, manuals and instructional videos were available on the official website [10], and regional support for the implementation was provided by a network of 24 Olympic clubs in Slovakia, with members who were university graduates in PE assisting teachers upon request. For each student, standardized *z*-scores were used, allowing for comparisons across different regions, genders, and grade levels. The *z*-score calculation, representing the mean standardized score across all physical tests, was performed following the methodological framework established by Field [13]. The *Z*-scores were aggregated into 36 “large” districts, which exhibit better nodality compared to standard districts. The delimitation of large districts is shown in Fig. S1. Independent variables were also computed within the same spatial structure.

### Study variables

The study investigated regional differences in physical fitness by analyzing the influence of socioeconomic conditions, school infrastructure, and demographic health indicators [14]. Socioeconomic data included regional unemployment rates, social welfare dependency, and educational attainment levels. School infrastructure indicators considered the availability of gymnasiums and outdoor sports facilities, while gender-specific variables, such as wage disparity and gender-based educational gaps, were included in models analyzing female students. The data on infrastructure and school sports facilities were obtained from the Feasibility Study on the Development of Gymnasiums and School Yards in Primary Schools [15]. Health-related variables encompassed average life expectancy and BMI. Body weight and height were collected during physical fitness assessment. Physical education teachers were overseen by seasoned examiners to prevent errors in data collection and measurement. Height was measured to the nearest 0.1 cm, and body weight was assessed to the nearest 0.1 kg while wearing minimal clothing and without shoes. BMI was calculated as weight in kilograms divided by height in meters squared.

### Data analysis

#### Factor analysis for predictor reduction

To reduce collinearity among explanatory variables, Principal Component Analysis with Varimax rotation was performed. The procedure for implementing factor analysis was based on the publication by Rabušic *et al*. [16] This process extracted four key factors:

- Social Vulnerability, representing unemployment rates, social welfare dependency, and early maternal age.
- Knowledge Capital, which encompassed educational attainment and household income levels.
- Family Composition, incorporating household size and the prevalence of single-parent households.
- Gender Equality, included exclusively in models assessing female students.

In addition to the four mentioned components, the following further predictors entered the regression analysis: average life expectancy, average BMI value for the respective gender and grade, and lack of school infrastructure.

#### Statistical models and analysis

To examine the impact of regional characteristics on physical fitness, four separate multiple linear regression models were developed, stratified by gender and age group: (i) Model A: boys, Grades 6–7 (ages 12–13 years), (ii) Model B: Boys, Grades 8–9 (ages 14–16 years), (iii) Model C: Girls, Grades 6–7 (ages 12–13 years), and (iv) Model D: Girls, Grades 8–9 (ages 14–16 years). The dependent variable in all models was overall physical fitness performance, calculated as the average *z*-score across EUROFIT test components. Model performance was evaluated using the coefficient of determination (*R*^2^), assessing explanatory power. To ensure robustness, a Variance Inflation Factor analysis was conducted to verify the absence of multicollinearity. The Kolmogorov–Smirnov test confirmed the normality of residuals, and homoskedasticity was assessed using the Breusch–Pagan test. Statistical significance was set at *P* < .05 for all analyses. All statistical analyses were performed using IBM SPSS Statistics software.

## Results

### Sample characteristics

After excluding children who did not provide informed written consent or valid identification, data from a total of 42 741 students from 465 public schools were analyzed.

BMI categories were adjusted by age- and sex-specific WHO BMI reference percentiles [17]. Across all grades, the majority of children were classified as having normal weight, with proportions increasing with age (boys: 48% in Grade 6 and 60% in Grade 9; girls: 55%–68%). The prevalence of overweight and obesity decreased across grades in both sexes. In boys, the combined proportion declined from 36% in Grade 6 to 29% in Grade 9, and in girls from 26% to 18%. The proportion of underweight remained relatively stable, ranging from 15% to 17% in boys and 14%–18% in girls. Detailed results are presented in Table S1.

To determine the physical fitness level, test scores were compared to the 50th percentile of European reference values [18] (Table 1). Girls exceeded reference values in cardiorespiratory fitness and speed/agility but scored lower in muscular power and endurance. Younger boys exceeded reference values in speed/agility but performed below the median in other domains, while older boys exceeded it in muscular power and upper-body endurance (bent arm hang). Across all grades, boys remained below the median in cardiorespiratory fitness. Sit-ups were the weakest domain for both sexes and all grades.

**Table 1.** Physical fitness of 42 741 primary school students in Grades 6–9 (ages 12–16 years), compared to European reference values

	Boys			
	Grade 6	Grade 7	Grade 8	Grade 9
	(*n* = 5848)	(*n* = 5741)	(*n* = 5832)	(*n* = 4912)
Bent arm hang (s)	7.0	9.9	14.9	20.0
Reference	11.2	12.0	12.5	12.8
Standing broad jump (cm)	160	171	188	202
Reference	170	175	180	185
10 × 5 m shuttle run (s)	20.8	20	19.3	18.9
Reference	20.1	19.8	19.5	19.3
Sit-ups (30 s) (reps)	23	24	26	27
Reference	33	35	36	37
Endurance shuttle run (laps)	32	39	44	50
Reference	43	47	50	51

	Girls			
	Grade 6	Grade 7	Grade 8	Grade 9
	(*n* = 5569)	(*n* = 5401)	(*n* = 5248)	(*n* = 4190)
Bent arm hang (s)	5.0	5.6	5.7	6.1
Reference	8.4	8.6	8.7	8.9
Standing broad jump (cm)	152	160	163	167
Reference	160	165	170	175
10 × 5 m shuttle run (s)	21.6	21.0	20.8	20.6
Reference	21.0	20.7	20.5	20.3
Sit-ups (30 s) (reps)	21	22	22	22
Reference	30	31	32	33
Endurance shuttle run (laps)	24	27	28	28
Reference	23	25	27	27

Observed and reference values represent the 50th percentile.

**Figure 1.**
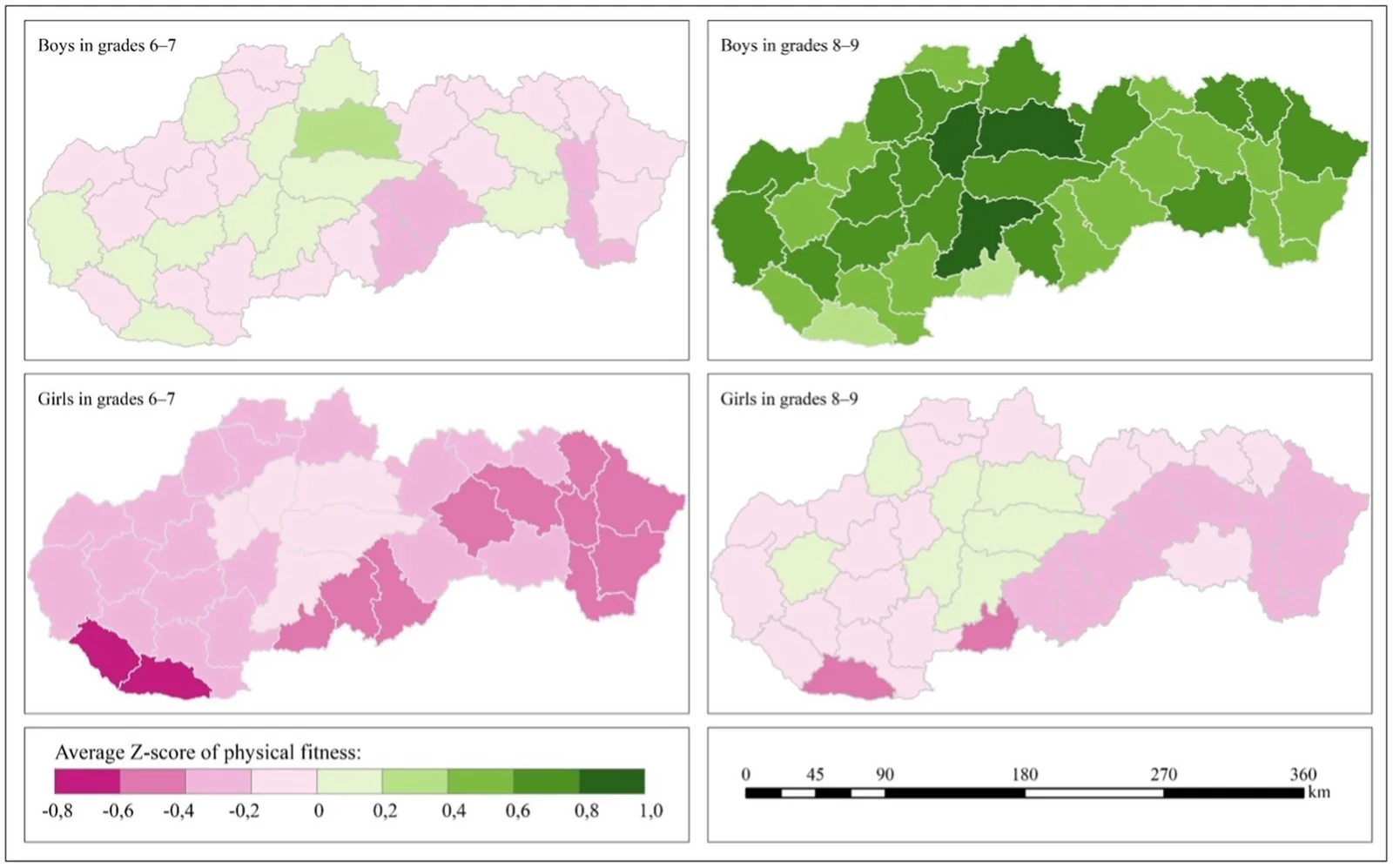
Average *Z*-score of physical fitness in Slovak regions.

**Figure 2.**
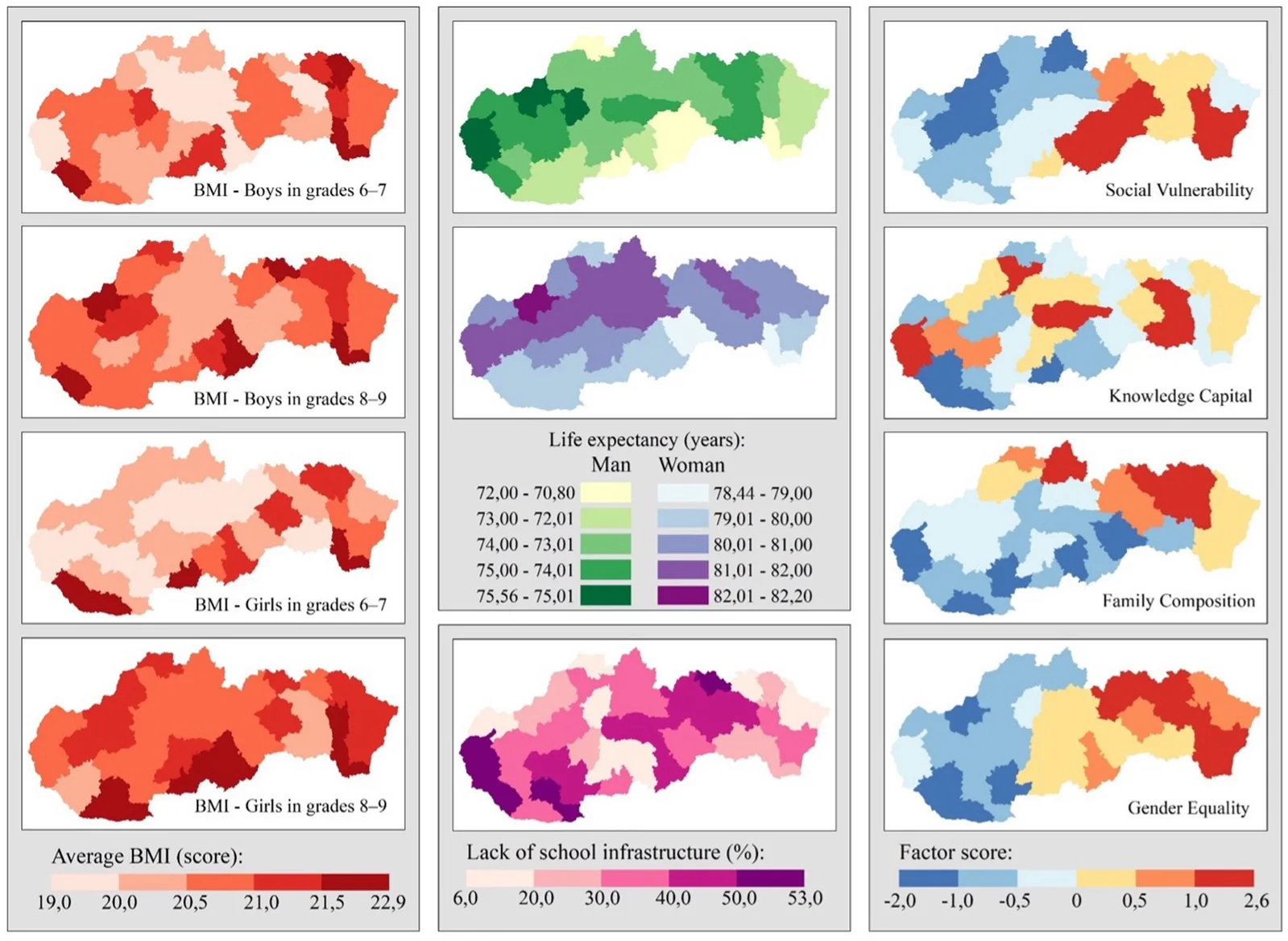
Spatial distribution of predictors.

The regression analysis examined the determinants of physical fitness in school children across four models, stratified by gender and school grade level. For dependent variables in regression models, the average *Z*-score of physical fitness in Slovak regions was used. The spatial distribution of this indicator is shown in Fig. 1. The models evaluated the influence of socioeconomic inequalities, school infrastructure, health indicators, and social determinants on overall physical fitness performance. The spatial distribution of predictors is shown in Fig. 2.

### Physical fitness predictors

**Figure 3.**
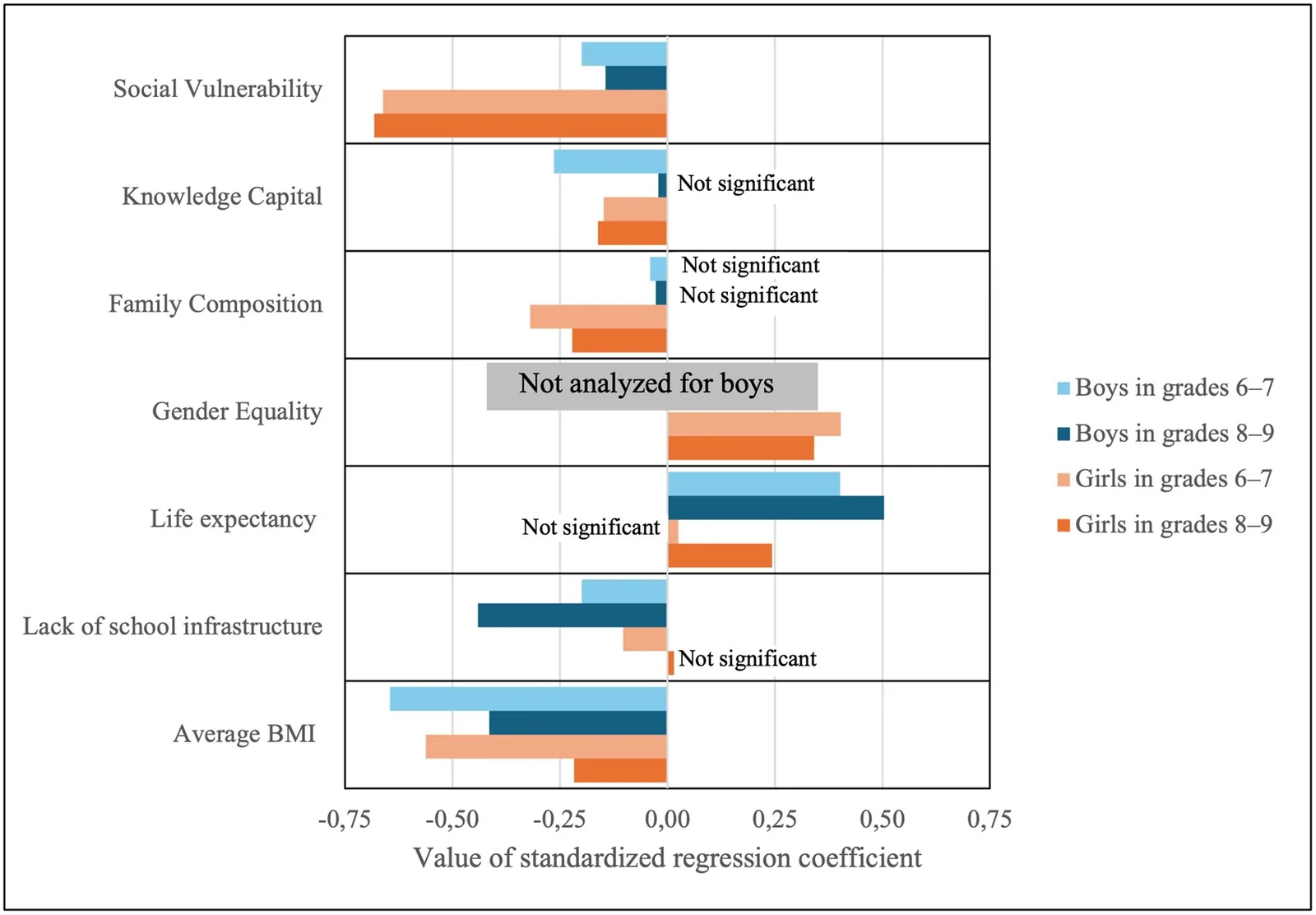
Standardized regression coefficients in analyzed models.

The coefficient of determination (*R*^2^) varied between 0.58 and 0.71, indicating strong explanatory power across models. Fig. 3 presents the standardized regression coefficients analyzed across all models. A comprehensive analysis of all models is provided in Table S2.

#### Physical fitness predictors in boys

For boys in Grades 6–7 (Model A), all predictors were statistically significant (*P* < .05), explaining 58% of the variance in physical fitness performance (*R*^2^ = 0.58). The most influential predictor was BMI, with a standardized regression coefficient of −0.64, indicating a strong negative relationship between higher BMI and lower physical fitness. Average life expectancy showed a significant positive effect (*β* = 0.40), suggesting that boys in regions with higher life expectancy exhibited better physical fitness scores. Additionally, social vulnerability (*β* = −0.20), lack of sports infrastructure (*β* = −0.20), and knowledge capital (*β* = −0.26) were negatively associated with physical fitness, indicating that poorer socioeconomic inequalities and insufficient school facilities contribute to lower physical fitness levels.

For boys in Grades 8–9 (Model B), the factors of knowledge capital and family composition turned out to be insufficiently significant. However, explanatory power increased to 63% (*R*^2^ = 0.63). The strongest predictor remained BMI (*β* = −0.41), while average life expectancy (*β* = 0.50) continued to positively influence physical fitness. In contrast to younger boys, the impact of school infrastructure became more pronounced, with inadequate sports facilities significantly reducing physical fitness (*β* = −0.44). Social vulnerability maintained a negative association (*β* = −0.14), further emphasizing the role of socioeconomic inequalities in shaping fitness outcomes.

#### Physical fitness predictors in girls

In the models for girls, the gender equality factor is additionally included among the predictors. For girls in Grades 6–7 (Model C), the explanatory power was higher than for boys (*R*^2^ = 0.66). Unlike boys, social vulnerability emerged as the most significant predictor (*β* = −0.66), suggesting that socioeconomic inequalities disproportionately impact girls’ physical fitness. BMI (*β* = −0.56) remained a strong negative predictor, but average life expectancy did not significantly contribute (*P* > .1). Instead, gender equality (*β* = 0.40) became a notable positive predictor, indicating that girls in regions with higher employment and educational equity achieved better physical fitness results. Additionally, family composition (*β* = −0.32) showed a significant negative effect, with larger families correlating with lower physical fitness.

For girls in Grades 8–9 (Model D), the overall explanatory power reached 71% (*R*^2^ = 0.71), the highest among all models. The effect of social vulnerability further intensified (*β* = −0.68), reinforcing its strong negative impact on girls’ physical fitness. Gender equality (*β* = 0.34) remained a significant positive predictor, while BMI (*β* = −0.22) and average life expectancy (*β* = 0.24) demonstrated moderate influences. Family composition (*β* = −0.22) continued to negatively affect fitness outcomes. Interestingly, school infrastructure was not statistically significant (*P* = .49) for older girls, suggesting that access to sports facilities played a lesser role in their physical fitness compared to boys.

## Discussion

In this cross-sectional study, we performed the regression analysis to evaluate four distinct models based on student groupings: boys and girls in Grades 6–7 (Models A and B) and boys and girls in Grades 8–9 (Models C and D) to observe insights into the contributions of various factors—such as social vulnerabilities, sport infrastructure, and health-related determinants—to the dependent variable. We hypothesized that sociodemographic and socioeconomic factors would significantly influence the physical fitness of school children, with the strength and direction of these influences varying by gender. We also hypothesized that school infrastructure would be associated with physical fitness disparities among school children, with gender-specific and age-related differences in their effects. Finally, we presumed that health-related variables show predictive power for physical fitness in gender-specific differences. The main findings of the study are: (i) the social vulnerability factor had a negative association with the physical fitness across all models, (ii) gender equality appeared to be a strong predictor for both younger and older girls, (iii) the higher knowledge capital component was associated with lower physical fitness, especially in younger students, (iv) health-related variables were more predictive in models for boys, and (v) infrastructure-related variables were negatively associated with the physical fitness but chiefly in boys.

Our results demonstrate that higher social vulnerability of regions is associated with poorer physical fitness in both boys and girls. This finding is consistent with research indicating that regions with higher unemployment rates and greater social welfare dependency tend to have lower levels of physical activity, influencing physical fitness among children and adolescents [19]. Possible explanations for the findings include increased parental psychosocial stress or a reduced capacity to support children’s active lifestyles. Chronic stress from unemployment or welfare dependence can lead to unhealthy coping mechanisms, such as reduced physical activity and poor dietary habits, which may contribute to childhood obesity [20]. Furthermore, parents dealing with a high level of vulnerability might feel less confident or more helpless, making it harder to encourage their children to be physically active—a crucial factor for improving children’s physical fitness [21]. Notably, regional social vulnerability was the most significant factor in girls, while it played a less significant role for boys. This may be due to factors like early fertility, which tends to affect girls more. Additionally, greater gender equality emerged as one of the most powerful predictors in both younger and older girls. It is known that individuals residing in regions with greater gender equality in areas such as employment, wages, or education are more likely to report higher physical activity levels than those residing in countries characterized by low gender equality [22]. These findings highlight how social factors are crucial in influencing girls’ fitness, while economic factors seem to be more influential for boys.

Interestingly, our findings that higher knowledge capital of regions are associated with lower physical fitness challenge the commonly assumed positive relationship between socioeconomic status and physical fitness. Most studies have linked the regions with higher education and household income to improved overall health outcomes [23], as higher incomes are typically associated with better nutritional status, housing conditions, healthcare, and more. Furthermore, individuals with higher education tend to have greater health awareness and knowledge about the necessity of physical activity [24]. However, there is limited evidence supporting the opposite, as seen in our study. Wang *et al*. [25] examined that high socioeconomic status was positively associated with insufficient physical activity. One possible explanation is that families with higher education and household income prioritize academic success, which can unintentionally lead to reduced physical activity among children. The phenomenon known as “Hurried Child Syndrome” describes situations where children are pressured to grow up too quickly, often due to parental expectations, academic pressures, or societal influences [26]. This pressure can lead to overly packed schedules, limiting time for unstructured play and physical activity. On the other hand, children from lower socioeconomic backgrounds may engage in more physically demanding household tasks or active commuting to school, which could increase their physical activity levels [27]. Furthermore, lower-income neighborhoods are distinguished by a greater number of locations offering youth physical activity opportunities, such as faith-based organizations, courts, trails/paths, event spaces, and water-based amenities [28]. These findings suggest that while regional social vulnerability and gender inequality present clear disadvantages for physical fitness, higher knowledge capital within these regions does not necessarily translate into increased physical activity, particularly in younger boys and girls.

Furthermore, our findings are consistent with previous research, demonstrating that the health-related indices (average life expectancy of a community and BMI) are significant predictors of children’s physical fitness and activity [29]. Average life expectancy can reflect the overall health environment of a region, influencing children’s physical fitness through its association with health-related factors such as BMI and general well-being. Regions with higher average life expectancy often maintain better public health standards, such as lower rates of childhood obesity [30] and more robust healthcare systems [31]. A higher life expectancy in the community is more likely to encourage engagement in physical activities among children, fostering fitness-oriented habits that contribute to healthier BMI levels [32]. BMI plays a crucial role in physical activity levels, as demonstrated also in our research. Excess body weight imposes both mechanical and metabolic strain, making movement more difficult and increasing the energy required for physical tasks. This discourages exercise, leading to lower physical activity levels. Furthermore, underperformance in physical activities can negatively impact emotional well-being, reducing self-confidence and potentially triggering anxiety or depression, which further diminishes motivation for activity [33]. On the other side, initial physical inactivity contributes to weight gain [34], which forms a vicious cycle. These interconnected factors explain why health-related indices are a strong predictor of physical fitness.

Moreover, most studies have reported these findings, especially in boys, which aligns with our results [35]. In boys, BMI was identified as the most powerful predictor of physical fitness, whereas in girls, BMI was the second most important factor, following social variables. These observed gender differences can be explained by physiological and behavioral variations. Boys usually have more lean muscle mass and higher testosterone levels [36], which increase significantly around the age of 11. These physiological factors contribute to enhanced muscle development and distinct physical activity patterns, thereby amplifying the role of BMI in determining physical fitness. Furthermore, boys are more likely to participate in high-intensity or competitive sports, where health metrics like BMI play a critical role in performance outcomes [37]. In contrast, girls tend to engage in less physically demanding activities, reducing the influence of BMI as a predictor of fitness. Nevertheless, BMI remains an important factor for girls.

Lastly, our findings support the importance of the school infrastructure-related variables in regions, as was demonstrated in a positive association with the physical fitness of both younger and older boys. Specifically, factors such as the availability of school environments or outdoor sports fields and their quality have been shown to positively influence physical fitness, particularly cardiovascular fitness and muscular endurance [38]. As we mentioned, the positive association of infrastructure-related variables with physical fitness was demonstrated, especially in boys. It is well-established that boys practice more physical activity and prefer team sports like football, which require specific infrastructure. In contrast, girls tend to engage in less infrastructure-intensive activities, such as walking, stretching, or dancing, which could dilute the impact of the school environment on their physical fitness [39]. Moreover, studies have demonstrated that greater space per student is associated with higher activity levels, highlighting the necessity of adequate infrastructure. Additionally, the accessibility of facilities in extracurricular opportunities significantly contributes to adolescents’ physical activity levels [38]. A lack of motivating school environments has been reported as one of the main reasons for insufficient physical activity during school hours, which underscores the importance of creating engaging and supportive spaces [40].

A key strength of our research is its distinct approach to socioeconomic determinants. Rather than combining indicators, we examined unemployment and social welfare dependency separately as markers of social vulnerability. The study also contributes to limited evidence on links between parental gender equality and children’s physical fitness. Additional strengths include a large, nationally representative sample and analyses stratified by gender and age. Limitations include the cross-sectional design, exclusion of behavioral factors, and the use of regional rather than individual socioeconomic data. Psychological factors were not assessed, and school infrastructure was evaluated only in terms of availability, not quality or accessibility. Although all PE teachers were university graduates and formally trained, minor variation in test implementation cannot be fully excluded. Despite these constraints, the study identifies key predictors of fitness disparities and offers direction for further policy interventions.

## Conclusion

This study highlights the complex interplay of socioeconomic, health, and environmental factors in shaping physical fitness among school children in Slovakia. BMI and social vulnerability emerged as the strongest negative predictors, while life expectancy and school infrastructure positively influenced fitness, particularly in boys. Gender-specific differences indicate that socioeconomic disadvantages disproportionately impact girls, whereas BMI plays a more critical role in boys’ fitness outcomes. The findings underscore the need for targeted interventions addressing obesity prevention, socioeconomic disparities, and school facility improvements. Future research should incorporate longitudinal analyses and behavioral factors to further refine our understanding of these determinants.

## Supplementary Material

ckag016_Supplementary_Data

## Data Availability

Results of all analyses are included in this published article. The datasets generated and/or analyzed during the current study are available from the corresponding author on reasonable request. Key points Socioeconomic disadvantage negatively influenced physical fitness, with a stronger impact on girls than boys. Economic hardship and early-life challenges contributed significantly to lower physical fitness levels, particularly in older age groups. School sports infrastructure played a crucial role in determining physical fitness, especially among boys in older grades. Limited access to sports facilities and extracurricular activities was associated with reduced physical fitness outcomes. Regional health indicators, particularly life expectancy, were positively associated with physical fitness. This suggests that broader health conditions within a region may influence children’s physical activity and overall fitness levels.
