# Supplementary material for: Regional, socioeconomic, and health determinants of physical fitness in school children: insights from a National Olympic Fitness Project
Source: Eur J Public Health. 2026 Feb 26;36(3):ckag016. doi: 10.1093/eurpub/ckag016 (PMC13230499; doi:10.1093/eurpub/ckag016)
Supplement: ckag016_Supplementary_Data [file ckag016_supplementary_data.zip › ejph-2025-02-om-0139-File006.docx]

**Table S1** BMI of 42,741 primary school students in grades 6 to 9 (ages 12–16 years).

|  | **Boys** | | | |
| --- | --- | --- | --- | --- |
|  | **Grade 6** | **Grade 7** | **Grade 8** | **Grade 9** |
| **Underweight** | 905 (15%) | 955 (17%) | 853 (15%) | 557 (11%) |
| **Normal weight** | 2821 (48%) | 2879 (50%) | 3269 (56%) | 2939 (60%) |
| **Overweight** | 1106 (19%) | 1054 (18%) | 950 (16%) | 887 (18%) |
| **Obesity** | 1016 (17%) | 853 (15%) | 758 (13%) | 527 (11%) |
|  | **Girls** | | | |
|  | **Grade 6** | **Grade 7** | **Grade 8** | **Grade 9** |
| **Underweight** | 1028 (18%) | 908 (17%) | 777 (15%) | 582 (14%) |
| **Normal weight** | 3059 (55%) | 3204 (59%) | 3359 (64%) | 2855 (68%) |
| **Overweight** | 907 (16%) | 836 (15%) | 739 (14%) | 520 (12%) |
| **Obesity** | 568 (10%) | 451 (8%) | 371 (7%) | 232 (6%) |

Values are presented as the number of children and the percentage of the total sample within each grade. BMI categories were adjusted by age- and sex-specific WHO BMI reference percentiles ^1^

References

1 World Health Organization. BMI-for-age (5-19 years). 2025.URL https://www.who.int/tools/growth-reference-data-for-5to19-years/indicators/bmi-for-age.
