# Supplementary material for: Regional, socioeconomic, and health determinants of physical fitness in school children: insights from a National Olympic Fitness Project
Source: Eur J Public Health. 2026 Feb 26;36(3):ckag016. doi: 10.1093/eurpub/ckag016 (PMC13230499; doi:10.1093/eurpub/ckag016)
Supplement: ckag016_Supplementary_Data [file ckag016_supplementary_data.zip › ejph-2025-02-om-0139-File007.docx]

Table S2: The comprehensive data analysis across all models.

| Model variant | Model A | | | | | | | Model B | | | | | | |
| --- | --- | --- | --- | --- | --- | --- | --- | --- | --- | --- | --- | --- | --- | --- |
| Dependent Variable | Average Z-score of physical fitness - Boys in grades 6–7 | | | | | | | Average Z-score of physical fitness - Boys in grades 8–9 | | | | | | |
| R^2^ | 0.58 | | | | | | | 0.63 | | | | | | |
| Predictors | Coefficients | | | | | t | Sig. | Coefficients | | | | | t | Sig. |
|  | Unstandard. | Std. Error | 95% Conf. Interval | | Standard. |  |  | Unstandard. | Std. Error | 95% Conf. Interval | | Standard. |  |  |
|  |  |  | Lower | Upper |  |  |  |  |  | Lower | Upper |  |  |  |
| Constant | 0.31 | 0.19 | -0.05 | 0.67 |  | 1.67 | 0.09 | -0.16 | 0.23 | -0.61 | 0.30 |  | -0.68 | 0.50 |
| Social Vulnerability | -0.02 | 0.00 | -0.03 | -0.02 | -0.20 | -7.94 | 0.00 | -0.02 | 0.00 | -0.02 | -0.01 | -0.14 | -6.45 | 0.00 |
| Knowledge Capital | -0.02 | 0.00 | -0.02 | -0.01 | -0.26 | -6.30 | 0.00 | 0.00 | 0.00 | -0.01 | 0.00 | -0.02 | -0.60 | 0.55 |
| Family Composition | 0.00 | 0.00 | -0.01 | 0.00 | -0.04 | -1.66 | 0.10 | 0.00 | 0.00 | -0.01 | 0.00 | -0.03 | -1.17 | 0.24 |
| Life expectancy (Man) | 0.03 | 0.00 | 0.03 | 0.04 | 0.40 | 12.05 | 0.00 | 0.05 | 0.00 | 0.04 | 0.05 | 0.50 | 16.71 | 0.00 |
| Lack of school infrastructure | 0.00 | 0.00 | 0.00 | 0.00 | -0.20 | -8.50 | 0.00 | 0.00 | 0.00 | 0.00 | 0.00 | -0.44 | -20.00 | 0.00 |
| Average BMI (Boys in grades) | -0.12 | 0.01 | -0.13 | -0.11 | -0.64 | -23.61 | 0.00 | -0.12 | 0.01 | -0.13 | -0.11 | -0.41 | -18.83 | 0.00 |
|  | | | | | | | | | | | | | | |
| Model variant | Model C | | | | | | | Model D | | | | | | |
| Dependent Variable | Average Z-score of physical fitness - Girls in grades 6–7 | | | | | | | Average Z-score of physical fitness - Girls in grades 8–9 | | | | | | |
| R^2^ | 0.66 | | | | | | | 0.71 | | | | | | |
| Predictors | Coefficients | | | | | t | Sig. | Coefficients | | | | | t | Sig. |
|  | Unstandard. | Std. Error | 95% Conf. Interval | | Standard. |  |  | Unstandard. | Std. Error | 95% Conf. Interval | | Standard. |  |  |
|  |  |  | Lower | Upper |  |  |  |  |  | Lower | Upper |  |  |  |
| Constant | 1.64 | 0.42 | 0.81 | 2.46 |  | 3.88 | 0.00 | -2.26 | 0.40 | -3.05 | -1.48 |  | -5.61 | 0.00 |
| Social Vulnerability | -0.08 | 0.01 | -0.09 | -0.07 | -0.66 | -14.92 | 0.00 | -0.10 | 0.00 | -0.10 | -0.09 | -0.68 | -24.75 | 0.00 |
| Knowledge Capital | -0.01 | 0.00 | -0.02 | -0.01 | -0.15 | -4.28 | 0.00 | -0.01 | 0.00 | -0.02 | -0.01 | -0.16 | -4.85 | 0.00 |
| Family Composition | -0.03 | 0.00 | -0.04 | -0.03 | -0.32 | -11.45 | 0.00 | -0.02 | 0.00 | -0.03 | -0.02 | -0.22 | -9.23 | 0.00 |
| Gender Equality | 0.05 | 0.00 | 0.04 | 0.06 | 0.40 | 10.00 | 0.00 | 0.00 | 0.00 | 0.00 | 0.01 | 0.34 | 14.44 | 0.00 |
| Life expectancy (Woman) | 0.00 | 0.00 | -0.01 | 0.01 | 0.03 | 0.75 | 0.46 | 0.04 | 0.00 | 0.03 | 0.05 | 0.24 | 7.92 | 0.00 |
| Lack of school infrastructure | 0.00 | 0.00 | 0.00 | 0.00 | -0.10 | -4.52 | 0.00 | 0.00 | 0.00 | 0.00 | 0.00 | 0.02 | 0.70 | 0.49 |
| Average BMI (Girls in grades) | -0.11 | 0.01 | -0.12 | -0.10 | -0.56 | -21.53 | 0.00 | -0.06 | 0.01 | -0.07 | -0.05 | -0.22 | -9.73 | 0.00 |
