# Supplementary material for: Regional, socioeconomic, and health determinants of physical fitness in school children: insights from a National Olympic Fitness Project
Source: Eur J Public Health. 2026 Feb 26;36(3):ckag016. doi: 10.1093/eurpub/ckag016 (PMC13230499; doi:10.1093/eurpub/ckag016)
Supplement: ckag016_Supplementary_Data [file ckag016_supplementary_data.zip › ejph-2025-02-om-0139-File005.docx]

Figure S1: Delimitation of 'large' districts of the Slovak Republic ^1^


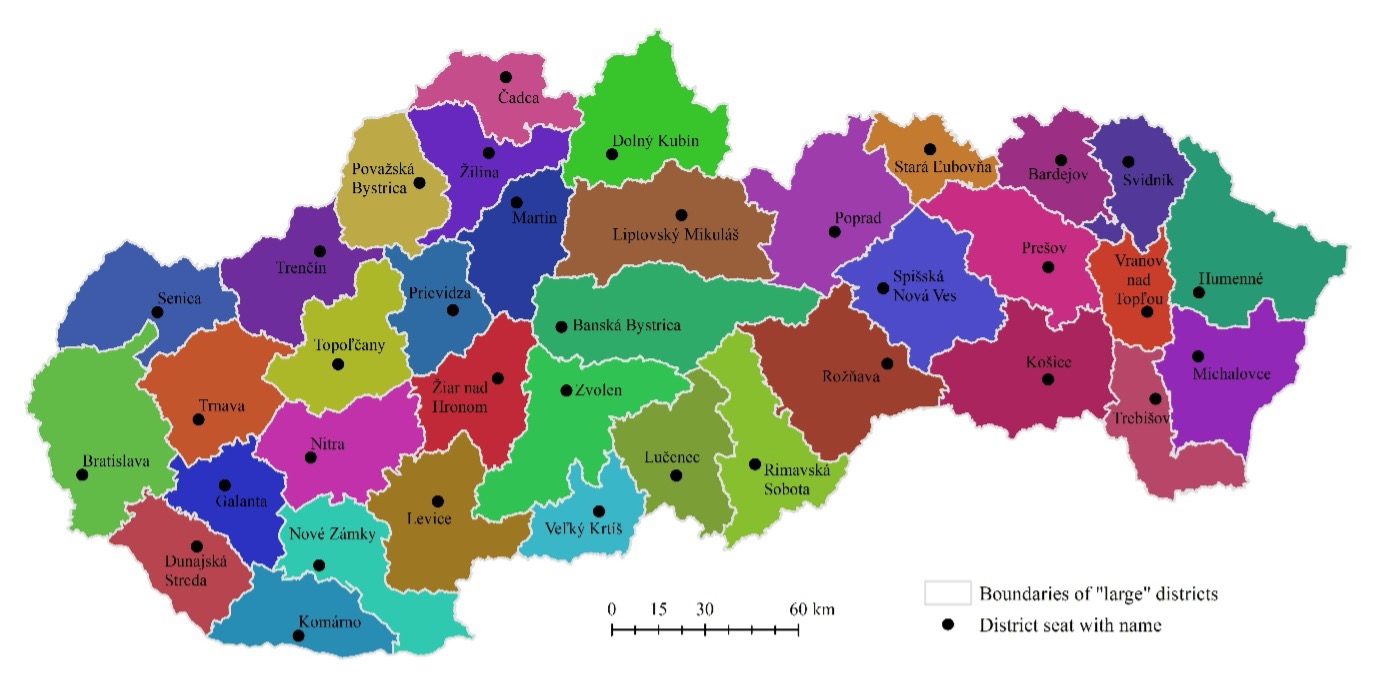


References:

1 Sociálna poisťovňa. Kontakty. Retrieved from Pobočky . 2024;12.URL https://www.socpoist.sk/po.
